# Association Between the Site of Infection and Mortality Analysis in Critically Ill Surgical Patients

**DOI:** 10.7759/cureus.50033

**Published:** 2023-12-06

**Authors:** Waleed Bin Ghaffar, Sidra Nazir, Summiya Siddiqui, Moeed B Abdul Ghaffar, Muhammad F Khan, Asad Latif, Zahra Cheema, Sadaf Hanif, Mohammad Sohaib

**Affiliations:** 1 Anaesthesiology, Aga Khan University Hospital, Karachi, PAK; 2 Surgery, Aga Khan University Hospital, Karachi, PAK; 3 Medicine, Aga Khan University Hospital, Karachi, PAK

**Keywords:** developing countries, mortality, intensive care units, surgical wound infection, intraabdominal infections, infections, septic shock, sepsis

## Abstract

Background

Sepsis remains a critical global health concern, leading to a high mortality rate. Existing literature suggests a potential correlation between infection site and mortality. Mortality data from Pakistan, especially in the context of the infection site, is notably scarce.

Purpose

The study aimed to explore the relationship between the site of infection and clinical features in deceased septic patients in the surgical intensive care unit.

Methods

In this retrospective study conducted at the Aga Khan University Hospital, data from patients admitted to the surgical intensive care unit and meeting Sepsis 3 criteria over a five-year period (2016-2020) were analyzed. We analyzed the relation between the infection site and clinical characteristics using analysis of variance (ANOVA), chi-square, or Fisher's exact tests. Multivariable logistic regression models were applied using stepwise forward selection. A p-value of ≤ 0.05 was considered statistically significant.

Results

A total of 2472 ICU admissions were screened, out of which 170 patients were included in this study. Demographic analysis showed a predominantly male population with a mean age of 47 years. The most prevalent site of infection was the abdomen. Logistic regression analysis identified on-admission septic shock and high Acute Physiology and Chronic Health Evaluation (APACHE) II scores as significant risk factors for 48-hour mortality while colistimethate sodium usage and admission through the operating room were protective.

Conclusion

Our study provides a comprehensive analysis, outlining infection sites and identifying early mortality-influencing factors within our region. The distinct demographic profile, characterized by younger age, and the prevalence of abdominal infections in the Pakistani cohort contradict established medical literature. Early initiation of broad-spectrum antibiotics, coupled with prompt source control, confers a protective effect upon individuals afflicted with sepsis.

## Introduction

Sepsis, a medical emergency, continues to be a leading factor of mortality in critically ill patients [1]. Acknowledging its worldwide significance, the World Health Assembly and the World Health Organization made sepsis a global priority in 2017 [2]. Despite advanced critical care management in the era of modern medicine, the worldwide mortality rates due to sepsis still range as high as 30% to 50% [3]. In resource-deprived regions, this scenario worsens, leading to a mortality rate of up to 80% [4]. Low and middle-income countries (LMIC) face particular challenges, including high disease burden, malnutrition, limited access to healthcare, and inadequate infrastructure [5]. These create notable differences in outcomes when compared to developed countries.

Literature elucidates that the systemic inflammatory response, activated by uncontrolled infection from an infection site, is the major factor contributing to mortality in septic shock [1]. Consequently, the anatomical origin of infection may impact patient progression and clinical outcome. Some studies have suggested a potential correlation between the site of infection and mortality outcomes [6-9]. The variation in infection sites and resulting outcomes can be attributed to some infection sites that facilitate rapid dissemination, causing early hemodynamic disturbances. However, others have refuted it, finding no association between the infection site and outcome [3,10]. Moreover, mortality data from LMIC is notably scarce. This data is essential for prioritizing patients and allocating resources. This information guides clinicians in making treatment decisions, facilitating evidence-based guidelines, and ultimately improving patient care.

In LMIC, the scarcity of morality data poses a significant challenge in drawing definitive conclusions and formulating effective treatment strategies for septic patients. Particularly, the literature exploring the relationship between the site of infection and its outcome, especially in developing countries like Pakistan, is notably limited. This lack of data highlights the critical necessity for comprehensive research initiatives in this field to guide healthcare strategies and improve patient outcomes.

The primary objective of this study was to evaluate the correlation between the site of infection and distinctive clinical traits in deceased patients suffering from sepsis or septic shock admitted to the surgical intensive care unit. The secondary objective was to investigate the correlation between the infection site and its association with the duration of survival in the same population.

## Materials and methods

This retrospective cross-sectional study was conducted at Aga Khan University Hospital, which is affiliated with a 664-bed capacity and a 15-bed intensive care unit (ICU). Data collection commenced after approval from the ethical review committee (ERC number 2020-5378-14600) of the hospital. Data for all patients admitted from January 1, 2016, to December 31, 2020, was extracted from the ICU registry. Patients meeting the Sepsis 3 criteria for sepsis or septic shock upon ICU admission were identified from hospital records. The hospital's health management information system was utilized to confirm these patients and corresponding file records were accessed and reviewed. We included patients presenting with on-admission sepsis or septic shock, characterized by the Sepsis 3 criteria, who succumbed within the intensive care unit (ICU) during the specified study period. Patients with missing medical files and restricted management directives (do not resuscitate orders) were excluded from the study.

Patient demographics, including age, gender, and comorbidities, along with the mode of admission and admission diagnosis, were recorded. The data were utilized to calculate the Charlson Comorbidity Index (CCI). Information regarding infections such as primary infection sites and responsible organisms, was identified upon admission and during the ICU stay. Sepsis was defined according to the Sepsis 3 criteria as life-threatening organ dysfunction caused by a dysregulated host response to infection [11,12]. Septic shock was identified clinically by the need for vasopressors to maintain a mean arterial pressure of 65 mmHg or greater and a serum lactate level greater than 2 mmol/L (>18 mg/dL) in the absence of hypovolemia [11,12]. Clinical details were obtained from the ICU progress file and nursing sheet while patient investigations (including laboratory, radiology, and microbiology) and medication records were acquired through a hospital-based web portal known as Patient Care Inquiry. Data related to organ support and outcomes, such as mechanical ventilation, renal replacement therapy, vasopressor/inotropic support, and duration of ICU stay, were systematically documented. The ICU stay of all patients was tracked until mortality. Data were collected using a specialized form designed specifically for this study. The confidentiality of the patient was maintained in accordance with hospital ethics committee guidelines.

Statistical analysis

The data analysis was conducted using RStudio (version 4.1.2; Boston, USA). Normality assumptions were assessed for quantitative variables such as age and APACHE II scores upon admission. Based on the distribution of the data, we calculated the mean ± standard deviation (SD). For qualitative variables, including gender, mode of admission, admission diagnosis, and others, we determined frequencies and percentages. To investigate the relationship between the site of infection and clinical characteristics, we employed the analysis of variance (ANOVA) test for quantitative variables and chi-square or Fisher's exact test for qualitative variables. Multivariable logistic regression models with a stepwise forward selection method were performed to identify factors associated with 48-hour mortality. A significance level of P ≤ 0.05 was considered statistically significant.

## Results

The ICU registry included data from 2472 surgical ICU admissions, with 341 patients experiencing mortality. Among these cases, sepsis was diagnosed on admission in 201 cases. After excluding 31 cases unrelated to sepsis mortality and the unavailability of records, data from a cohort of 170 patients was analyzed. Basic demographics and clinical characteristics are summarized in Table 1. The mean age was 47 (±17.2), with a male majority (67.6%). Postoperative cases constituted 46.5% of ICU admissions, with general surgery being the predominant specialty (68.8%).

**Table 1:** Demographics and clinical characteristics *Mean±SD: quantitative variables; †n (%): qualitative variables Abbreviations: APACHE, Acute Physiology and Chronic Health Evaluation

Variables	Estimates
Age (years)^*^	47.8 ± 17.2
Gender	
Male^†^	115 (67.6%)
Female^†^	55 (32.4%)
APACHE II on admission^*^	24.9 ± 9.08
Charlson Comorbidity Index^*^	1.80 ± 1.97
Source of admission	
Emergency department^†^	38 (22.4%)
Operating room^†^	79 (46.5%)
Ward^†^	53 (31.2%)
Admission diagnosis	
Sepsis^†^	42 (24.7%)
Septic shock^†^	128 (75.3%)
Surgical specialty	
General surgery^†^	117 (68.8%)
Neurosurgery^†^	29 (17.1%)
Orthopedic surgery^†^	4 (2.35%)
Otorhinolaryngology^†^	5 (2.94%)
Plastic surgery^†^	2 (1.18%)
Urology^†^	1 (0.588%)
Vascular surgery^†^	7 (4.12%)
Infection sites on ICU admission	
Single site^†^	152 (89.4%)
Multiple sites^†^	18 (10.6%)
Laboratory markers	
Lactate (mmol/L)^*^	6.11 ± 4.57
WBC (× 10^9^/L)^*^	14.4 ± 10.2
Procalcitonin (ng/ml)^*^	31.1 ± 37.0
Creatinine (mg/dl)^*^	1.76 ± 1.37
Organ support	
Mechanical ventilation - invasive^†^	167 (98.2%)
Mechanical ventilation - noninvasive^†^	3 (1.76%)
Renal replacement therapy^†^	51 (30.0%)
Vasopressor support^†^	163 (95.88%)
Inotropic support^†^	66 (38.8%)
Complications and outcome	
Acute Respiratory Distress syndrome^†^	83 (48.8%)
Mortality within 48 hours of admission^†^	50 (29.4%)
ICU Stay^*^	6.42 ± 6.42

Table 2 compares site-specific infections and associated clinical characteristics. Abdominal infections emerged as the most prevalent site of infection. Genitourinary infections correlated with the highest mean lactate (8.90±5.58) and the highest proportion of patients experiencing septic shock (83.33%). All other sites except for central nervous system infections had a mean APACHE score of ≥ 24. Pulmonary infections were associated with an increased predisposition to acute respiratory distress syndrome (ARDS). Patients with ARDS demonstrated a pronounced need for cardiovascular support, encompassing vasopressors and inotropes. Genitourinary infections exhibited the highest mortality rate within the initial 48 hours.

**Table 2:** Clinical characteristics associated with the site of infection on admission *Mean±SD: quantitative variables; †n (%): qualitative variables Abbreviations: CNS, central nervous system; APACHE II, Acute Physiology and Chronic Health Evaluation; MDR, multidrug-resistant; ARDS, acute respiratory distress syndrome

Variables	Site of infection						
	Abdomen (N=70)	Soft Tissue (N=46)	Blood (N=27)	Pulmonary (N=22)	Genitourinary (N=6)	CNS (N=13)	Osteoarticular (N=5)
Admission characteristics							
APACHE II^*^	25.5±9.37	24.5±9.36	27.4±8.93	26.0±9.44	26.7±7.42	19.7±6.47	24.6±6.91
Lactate (mmol/L)^*^	7.09±4.73	5.58±4.29	4.84±4.65	5.73±4.50	8.90±5.58	3.32±2.75	5.35±4.34
Septic shock^†^	58 (82.85%)	38 (82.60%)	18 (66.66%)	16 (72.72%)	5 (83.33%)	4 (30.76%)	2 (40%)
Characteristics during ICU stay							
MDR organism^†^	22 (31.42%)	19 (41.30%)	8 (29.62%)	6 (27.27%)	1 (16.66%)	2 (15.38%)	1 (20%)
ARDS^†^	35 (50%)	21 (45.65%)	14 (51.85%)	16 (72.72%)	1 (16.66%)	6 (46.15%)	3 (60%)
Mechanical ventilation days^*^	5.26±4.66	6.02±5.96	5.15±4.37	8.82±8.40	3.33±4.80	5.15±2.58	3.00±1.41
Vasopressor support^†^	70 (100%)	44 (95.65%)	25 (95.59%)	22 (100%)	6 (100%)	10 (76.9%)	4 (80%)
Inotropic support^†^	27 (38.57%)	19 (41.30%)	7 (25.92%)	13 (59.09%)	2 (33.33%)	3 (23.07%)	1 (25 %)
Renal replacement therapy^†^	23 (32.85%)	16 (34.78%)	5 (18.51%)	8 (36.36%)	1 (16.66%)	1 (7.69%)	2 (40%)
Mortality within 48 hours of admission^†^	22 (31.42%)	14 (30.43%)	7 (12.72%)	6 (25.92%)	4 (66.66%)	1 (7.69%)	1 (20%)

Out of the 170 patients studied, 55 (32.35%) succumbed to mortality within the initial 48 hours while 115 (67.65%) experienced mortality beyond the 48-hour mark. Table 3 presents findings from forward stepwise logistic regression analysis regarding factors associated with mortality within 48 hours of admission. The septic shock on admission (P value 0.006) and a high APACHE II score (P value 0.034) were significantly associated with 48-hour mortality. Colistimethate sodium usage had a negative correlation (adjusted OR: 0.18; 95% CI: 0.06 - 0.53; P value 0.002), suggesting a protective effect. ARDS also exhibited a protective effect (adjusted OR: 0.18; 95% CI: 0.06 - 0.52; P value 0.034), potentially due to a delayed mortality pattern in this group. Patients admitted through the operating room with the initial source control of the infection site showed a significant 48-hour survival rate (adjusted OR: 0.22; 95% CI: 0.06 - 0.84; P value 0.027).

**Table 3:** Factors contributing to 48-hour mortality Abbreviations: ARDS, acute respiratory distress syndrome; PRBS, packed red blood cell; FFP, fresh frozen plasma; APACHE, Acute Physiology and Chronic Health Evaluation

Factors	Multivariable Analysis	
	Adj. OR (95% CI)	P value
Mortality within 48 hours of admission		
Septic shock on admission	9.75 (1.90 - 50.14)	0.006
Colistimethate Sodium	0.18 (0.06 - 0.53)	0.002
Transfusion of Blood products (PRBC/ FFP/ Platelet)	0.26 (0.06 - 1.07)	0.062
ARDS	0.18 (0.06 - 0.52)	0.002
APACHE II	1.06 (1.01 - 1.12)	0.034
Mode of admission		
Emergency Department	Ref.=1.00	
Operating room	0.22 (0.06 - 0.84)	0.027
Ward	0.96 (0.24 - 3.82)	0.950

**Figure 1:**
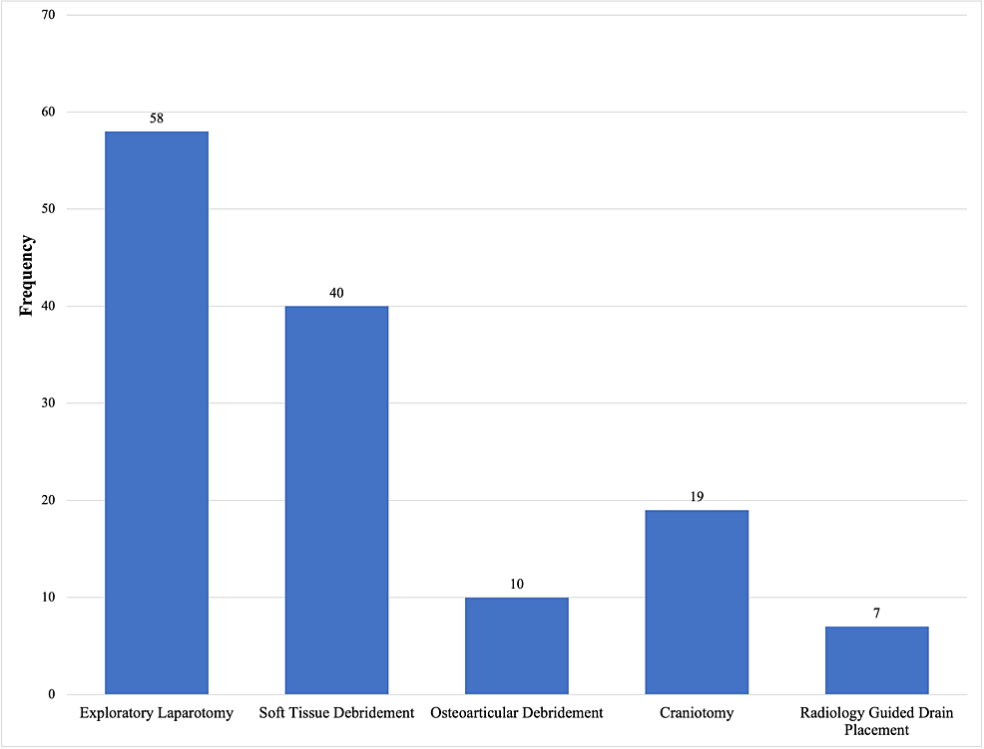
Surgical and radiology-guided interventions

We identified a total of 134 surgical and radiology-guided interventions that were performed either upon admission or during the ICU stay (Figure 1). These interventions were aimed at controlling the source of sepsis.

Carbapenems were the most prescribed initial antibiotics (32.3%), followed by vancomycin (30.7%). A substantial number (31.76%) of patients manifested multidrug-resistant (MDR) organisms, with Acinetobacter baumannii (42.4%) being the dominant pathogen. A variety of antifungal agents were administered, encompassing fluconazole, voriconazole, amphotericin B, and caspofungin, among which amphotericin B was the most prescribed antifungal (53.81%). However, fungal organisms were detected in 19.41% of patients.

## Discussion

Our demographic analysis indicated a male predominance of 67% with a mean age of 47 years. This relatively younger age average reflects the demographic pattern in Pakistan, where life expectancy is notably different from many reported datasets dominated by elderly populations in high to middle-income countries [3,6-9,13]. It’s noteworthy that 75% of our patients were admitted in a state of shock. This may reflect delays in patient presentation, a phenomenon often caused by economic constraints and limited healthcare accessibility, as seen in LMICs. The severity of illness at ICU admission was also evident by elevated lactate levels (6.11 mmol/L) and higher mean APACHE II scores (24.9) at admission.

In our research, the abdomen emerged as the predominant site of infection, constituting 41.2% of cases. This contrasts with prevailing literature, which typically designates the lungs as the most common primary infection site [3,6-9]. It’s noteworthy that our study, conducted exclusively in a surgical ICU, differs from previous ones that included both medical and surgical ICUs. The link between ICU mortality and abdominal infections may be attributed to the polymicrobial nature of these infections and the challenges associated with source control [14-16]. Morar et al. also demonstrated that delays in surgical intervention and inadequate source control during the initial laparotomy increased the risk of mortality in cases of abdominal sepsis [17].

Divergent findings regarding infection sites and mortality are evident in existing literature. Klastrup et al. suggested variability in site-specific mortality among severe septic patients. In contrast, Zahar et al. and Kumar et al. did not identify an independent association between site and mortality [3,6,18]. Our study, focusing solely on patients with mortality, precludes a direct comparison of site-specific mortality. Nevertheless, there were variations in the incidence of septic shock upon admission across different primary infection sites. Abe T et al. similarly observed that intra-abdominal infections were most frequently associated with septic shock, aligning with our results where septic shock occurred in 82.9% of patients with abdominal infections [8]. Patients admitted through the operating room demonstrated 48-hour survival, underscoring the pivotal importance of source control, a principle widely advocated in the literature [19-21].

Leligdowicz et al. identified the highest mortality rates in patients with ischemic bowel, gastrointestinal infection, and disseminated infection [7]. In our study cohort, individuals with genitourinary infections exhibited the highest severity, elevated lactate levels, an increased incidence of shock upon admission, and mortality within 48 hours of admission. The increased severity of genitourinary infections compared to abdominal infections may be attributed to the inclusion of patients with Fournier's gangrene within the genitourinary category, a condition associated with a mortality rate as high as 76% [22].

The study's patient population revealed that 95% required vasopressors, with 38.8% also requiring inotropes. Notably, despite abdominal infections being the predominant cause of sepsis (41.2%), a higher percentage of patients with pulmonary infections (59.1%) necessitated inotropic support in our study. Furthermore, patients with pulmonary infections experienced a higher prevalence of ARDS (72.72%) and endured the longest duration of ventilation days (8.82±8.40). This observation aligns with the established link between the duration of mechanical ventilation and adverse outcomes [23,24].

In our region, the prevalence of carbapenem-resistant Acinetobacter baumannii is on the rise [25]. This upward trend is reflected in our sample, where 31.8% of infections demonstrate MDR, with Acinetobacter baumannii accounting for 42.5% of these cases. This underscores the escalating issue of antibiotic resistance in our region, fueled by unrestricted access to antibiotics without prescriptions. Considering the uncertainty surrounding the causative organism during the initial presentation, our strategy involved initiating empirical, broad-spectrum antimicrobial coverage based on local colonization and resistance patterns [26-29]. Carbapenems were the most commonly prescribed initial antibiotics (32.3%), followed by vancomycin (30.7%). The increasing resistance pattern observed in MDR organisms prompted the empirical use of colistin in our center. Notably, the utilization of colistimethate sodium emerged as a protective factor against 48-hour mortality in our study. It is imperative to acknowledge the potential emergence of resistance in the near future, even to antibiotics like colistimethate sodium. The presence of MDR organisms intricately correlates with increased mortality [30].

In LMICs such as ours, there is a scarcity of data on critically ill patients. Conducting a retrospective analysis of 170 ICU mortalities, screened from a pool of 2400 critically ill intensive care admissions, provides valuable insights into surgical infection sites among this population. It's important to acknowledge the inherent limitations of retrospective studies, including potential data loss and recall bias. Prospective reviews of infection sites in deceased patients are not practically possible, underscoring the significance of retrospective analyses in shedding light on critical aspects of patient care in this high-risk group. Potential missed sites due to false-negative results cannot, however, be ignored. The study solely included patients with ICU mortality, omitting data on survivors. Nevertheless, the study's objective was to discern infection patterns within the high-risk subgroup of patients who did not survive, emphasizing the distinctive significance of this investigation. The study period coincided with the onset of the COVID-19 pandemic, necessitating acknowledgment of potential alterations in patient characteristics, emphasizing the need for careful analysis within this context. Our findings emphasize the significance of early intervention, as evident in the enhanced 48-hour survival rates observed among patients who underwent surgical source control via the operating room. Swiftly addressing infections by eliminating their source has demonstrated a decrease in the progression to shock and subsequent mortality [19-21].

## Conclusions

Our examination of 170 surgical ICU mortalities provided novel insights into caring for this high-risk group. Abdominal infections, in contrast to general literature, were most prevalent (41.2%) in this group. Logistic regression highlighted on admission septic shock and a high APACHE II score as significant risk factors for mortality within 48 hours while colistimethate sodium usage and admission through the operating room were protective. These findings underscore the importance of early intervention, tailored source control, and specific antimicrobial approaches in enhancing outcomes. Further studies are essential for refining strategies, particularly in resource-limited settings.
